# Experimental Investigation on Environmentally Sustainable Cement Composites Based on Wheat Straw and Perlite

**DOI:** 10.3390/ma15020453

**Published:** 2022-01-07

**Authors:** Andrea Petrella, Sabino De Gisi, Milvia Elena Di Clemente, Francesco Todaro, Ubaldo Ayr, Stefania Liuzzi, Magdalena Dobiszewska, Michele Notarnicola

**Affiliations:** 1 Dipartimento di Ingegneria Civile, Ambientale, del Territorio, Edile e Chimica, Politecnico di Bari, 4, Via E. Orabona, 70125 Bari, Italy; sabino.degisi@poliba.it (S.D.G.); milviaelena.diclemente@poliba.it (M.E.D.C.); francesco.todaro@poliba.it (F.T.); michele.notarnicola@poliba.it (M.N.); 2 Dipartimento di Scienze dell’Ingegneria Civile e dell’Architettura, Politecnico di Bari, 4, Via E. Orabona, 70125 Bari, Italy; ubaldo.ayr@poliba.it (U.A.); stefania.liuzzi@poliba.it (S.L.); 3 Faculty of Civil and Environmental Engineering and Architecture, Bydgoszcz University of Science and Technology, Al. Kaliskiego 7, 85-796 Bydgoszcz, Poland; magdalena.dobiszewska@pbs.edu.pl

**Keywords:** cement mortar, wheat straw, perlite, thermal insulation, acoustic absorption, secondary raw materials

## Abstract

Environmentally sustainable cement mortars containing wheat straw (Southern Italy, Apulia region) of different length and dosage and perlite beads as aggregates were prepared and characterised by rheological, thermal, acoustic, mechanical, optical and microstructural tests. A complete replacement of the conventional sand was carried out. Composites with bare straw (S), perlite (P), and with a mixture of inorganic and organic aggregates (P/S), were characterised and compared with the properties of conventional sand mortar. It was observed that the straw fresh composites showed a decrease in workability with fibre length decrease and with increase in straw volume, while the conglomerates with bare perlite, and with the aggregate mixture, showed similar consistency to the control. The thermal insulation of the straw mortars was extremely high compared to the sand reference (85–90%), as was the acoustic absorption, especially in the 500–1000 Hz range. These results were attributed to the high porosity of these composites and showed enhancement of these properties with decrease in straw length and increase in straw volume. The bare perlite sample showed the lowest thermal insulation and acoustic absorption, being less porous than the former composites, while intermediate values were obtained with the P/S samples. The mechanical performance of the straw composites increased with length of the fibres and decreased with fibre dosage. The addition of expanded perlite to the mixture produced mortars with an improvement in mechanical strength and negligible modification of thermal properties. Straw mortars showed discrete cracks after failure, without separation of the two parts of the specimens, due to the aggregate tensile strength which influenced the impact compression tests. Preliminary observations of the stability of the mortars showed that, more than one year from preparation, the conglomerates did not show detectable signs of degradation.

## 1. Introduction

Today, concern for environmental protection is growing, especially in the agro-food industry which generates wastes from direct consumption of primary products. Most of these by-products are non-hazardous and are currently underutilised or simply wasted. For this reason, the concept of bioeconomy is spreading as a new approach to production that gives new life to materials which would otherwise be destined for destruction [1,2,3,4,5,6,7,8]. Accordingly, the recycling of agro-food wastes as biofuels [9,10,11], fertilisers [12,13], energy [14], chemicals [15] and sorbents [16,17,18,19,20] is considered a valuable alternative to landfilling.

The main agricultural products in Italy include sugar beet, wheat, corn, tomatoes, oranges, potatoes, apples, barley, and rice. Therefore, a large amount of waste needs to be disposed of.

In the context of an environmental and sustainable approach, considerable effort is being invested in the exploitation of renewable cheap agricultural residues for the development of eco-building materials to limit greenhouse gas emissions, save natural resources and develop more energy efficient buildings [21]. The aim of bio-architecture is to construct healthy buildings with little ecological impact based on the use of sustainable, eco-friendly and cheap materials [7,22,23,24,25,26,27,28], such as cellulose fibres, which are among the most suitable secondary raw materials for this purpose. Specifically, wheat straw has been used as an aggregate in many low density and environmental safe, construction materials which have shown valuable mechanical, thermal and acoustic properties [22,23,24,29,30,31,32,33,34,35,36,37].

In this paper, eco-sustainable cement conglomerates, containing wheat straw from the Apulia region, Italy (33–38% cellulose, 26–32% hemicelluloses, 17–19% lignin [38]) and perlite beads, were prepared and characterised by rheological, thermal, acoustic, mechanical and microstructural measurements. A complete replacement of the conventional sand aggregate was carried out with straw cuttings of different length and dosage and with perlite beads. Composites with bare straw (S), perlite (P), and with a mixture of organic and inorganic aggregates (P/S), were characterised and compared with the properties of conventional sand mortar [39,40,41]. The addition of the perlite beads to the straw mixture was carried out to improve the mechanical properties of the conglomerates with little modification of the thermal insulation.

Many studies have considered the possibility of using specific treatments to prevent the degradation of this type of construction material resulting from the dissolution of the main constituents of the straw, ascribed to water absorption and alkaline pore solution [42,43,44,45]. Modification of the matrix composition, by addition of pozzolanic compound addition or by applying a carbonation process, was carried out to overcome the problems associated with the presence of alkaline compounds [46,47,48,49,50,51]. The stability of the natural fibres was improved by applying specific procedures, such as interface coatings, chemical structure modification, chemical products additions or combined processes [29,52,53,54,55,56,57,58,59,60,61].

The main purpose of the present research was to obtain eco-friendly thermo-insulating cement composites with natural and local by-products as aggregates for indoor applications [62,63,64,65]. The idea was to prepare and characterise these materials without the addition of fillers, additives, matrix modifiers or specific straw treatments, which, in some cases, are based on the use of reagents, such as silanes, gasoil, varnish, sodium hydroxide and sulfuric acid. In terms of the circular economy, these conglomerates were in accordance with current policies for environmental sustainability. Worthwhile processes were being carried out since these artifacts were prepared by a cheap process in which pre-treatment of renewable aggregates and complex or expensive procedures were not required [37,66,67,68,69,70].

## 2. Materials and Methods 

Cement mortars were prepared with CEM II A-LL 42.5 R [39] from Buzzi Unicem (Rc _(2 days)_ > 25.0 MPa, Rc _(28 days)_ > 47.0 MPa) which is characterised by 80–94% clinker, 6–20% limestone LL (<0.2% organic carbon), gypsum (0–5%), minor additional constituents, and which shows 3100–4400 cm^2^/g Blaine specific surface area. Natural wheat straw was used as total replacement of sand with cuttings (1.5–2.5 mm diameter) of variable length (0.4–0.6 cm, 1.3–1.7 cm, 3.4–3.7 cm, 5.8–6.2 cm).

Expanded perlite (P) (3–4 mm size range) is an inorganic material derived from volcanic rock with the following chemical composition: SiO_2_ 74.5%, Al_2_O_3_ 12.3%, K_2_O 4.2%, Na_2_O 4%, Fe_2_O_3_ 1%, CaO 1.4%. It was provided by Maltek Industrie S.r.l., Terlizzi, Bari, Italy. It is chemically inert, sterile and incombustible and with a granular form obtained after heat treatment at 760–1100 °C of the silica material which induces expansion [71,72].

**Figure 1:**
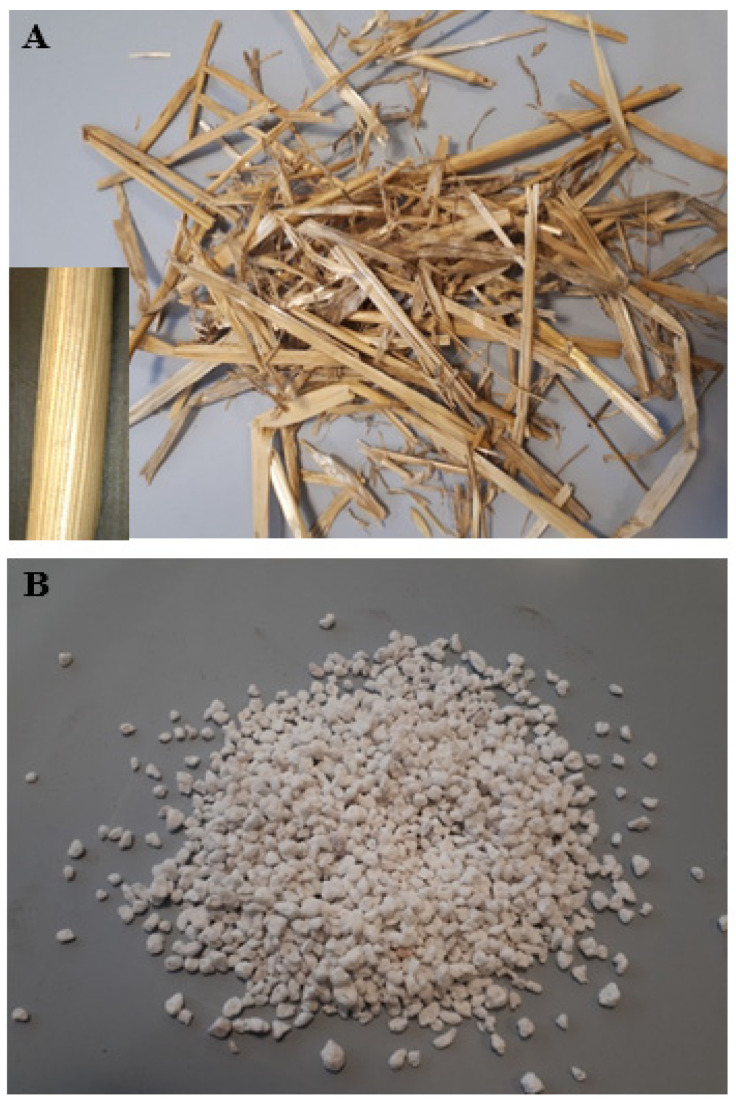
(**A**) Straw before use and, in the inset, after cutting. (**B**) Perlite beads.

**Figure 2:**
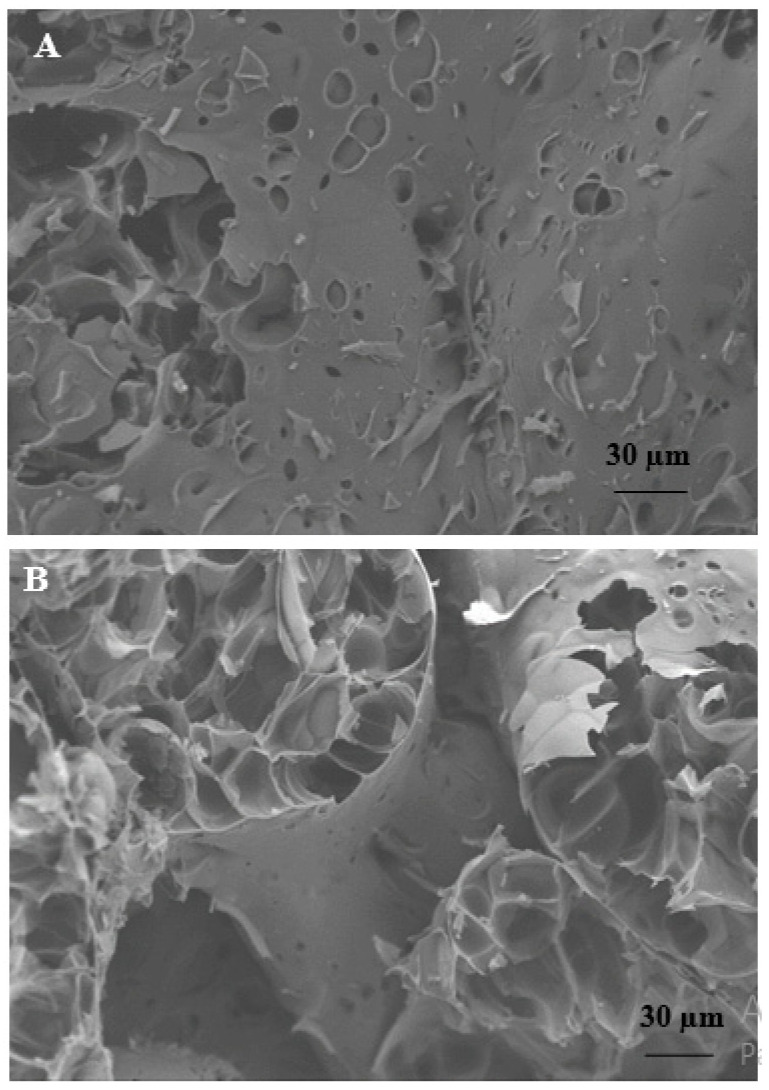
SEM image of (**A**) a perlite bead surface and (**B**) a perlite bead inner structure.

Figure 1A shows the straw before use and after cutting (see inset), while Figure 1B shows a picture of the perlite beads. Figure 2A shows an SEM image of a perlite bead surface, while Figure 2B shows its closed porosity.

In the case of the mortar preparation, a volume replacement of the conventional aggregate was carried out due to the lower density of the cellulose fibres and of the perlite beads with respect to sand [41]. The samples S1, S2, S3 and S4 were prepared with cuttings of different length (0.4–0.6 cm, 1.3–1.7 cm, 3.4–3.7 cm, 5.8–6.2 cm); the P sample was prepared with bare perlite, while the P/S1, P/S2, P/S3 and P/S4 samples were prepared with a mixture of perlite and straw. For all these samples, the volume of the aggregate was equal to 400 mL. The choice of this volume was a compromise to obtain comparable values for straw and perlite. Finally, S3A, S3B and S3C samples were prepared with the same straw length (3.4–3.7 cm) but with different volumes of aggregate (340 mL, 470 mL and 550 mL).

The conglomerates were prepared with tap water and with a water/cement ratio equal to 0.5 as in the case of conventional mortar preparation characterised by 225 g of water, 450 g of cement and 1350 g of normalised sand [40,73]. All the rheological, thermo-acoustic and mechanical measurements were compared with the results obtained with this normalised sand control. Table 1 reports the composition of all the mortars that were prepared for the present investigation.

The rheology of the fresh mixtures was evaluated using a flow-test [74]. Thermal and acoustic tests were carried out with cylindrical specimens (φ = 100 mm; H = 50 mm) after 28 days curing. Thermal measurements were based on the analysis of temperature response of dried specimens to heat flow impulses through a heating probe applied onto the surface of the sample [75]. For this purpose, an ISOMET 2104 device, from Applied Precision Ltd. (Bratislava, Slovakia), was used for the tests. An estimation of the thermal diffusivity (α) and thermal conductivity (λ) was obtained by comparison between the experimental temperature values and the analytical solution of the heat conduction equation. Acoustic absorption data were obtained after emission of a pure tone of known frequency at 250, 500, 1000 and 1600 Hz through a Kundt tube [76] characterised by a diameter sufficiently small with respect to the wavelength of the sound emission for stationary conditions measurements. A loudspeaker was positioned at one end of the tube and the sample placed at the other end.

Compression tests were carried out with a loading rate of 2400 ± 200 N/s on twelve semi-prisms which were obtained from flexural tests on six prisms (40 × 40 × 160 mm) at 50 ± 10 N/s loading rate [40]. For this purpose, a MATEST device, Milan, Italy, was used. In the case of the impact resistance tests, a steel ball (63 mm diameter) was placed on the upper surface of a specimen and a 4.50 kg weight was dropped from a height of 45 cm; after evaluation of the number of blows before fracture, the energy absorbed by the sample was obtained [77].

The aggregates and the conglomerates were also characterised by scanning electron microscope (SEM) and energy dispersive X-ray (EDX) analysis. For this purpose, an electron microscope FESEM-EDX Carl Zeiss Sigma 300 VP (Carl Zeiss Microscopy GmbH, Jena, Germany) was used after sputtering the samples with graphite (Sputter Quorum Q150 from Quorum Technologies Ltd., East Sussex, UK). The specimens were also characterised by an optical microscope (Dyno-lyte Digital Microscope, New Taipei City, Taiwan), while porosimetric measurements were carried out by Ultrapyc 1200e Automatic Gas Pycnometer (Quantachrome Instruments, Boynton Beach, FL, USA). For this, helium gas was used.

## 3. Results and Discussion

### 3.1. Rheological Tests

**Figure 3:**
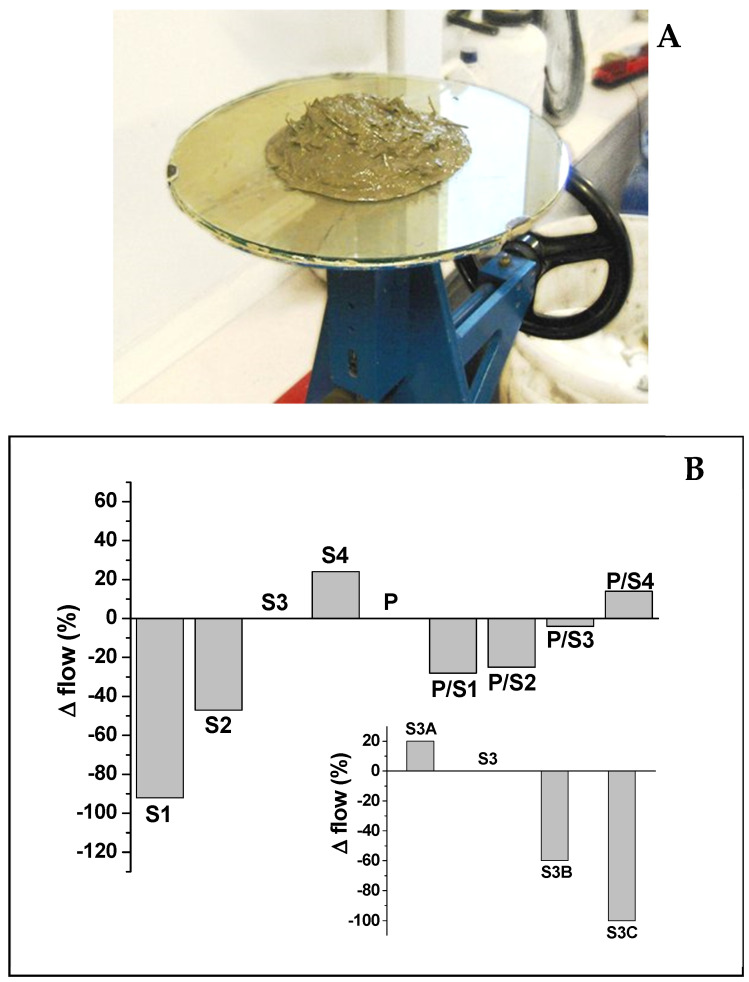
(**A**) Flow-test apparatus. (**B**) Flow-test results of the S, P and P/S samples with respect to the normalised mortar (Control).

The rheological tests carried out by the flow-test method enabled understanding of the flow and deformation of the fresh conglomerates, by modification of the aggregate composition and distribution, maintaining the same water and cement dosage. Figure 3 shows the workability of the non-consolidated specimens obtained with the flow-test in comparison with the conventional normalised sand mortar [74]. Fresh conglomerates with the least length of straw (S1 and S2) were drier than the normalised mortar (−90% and −50% respectively), with specific reference to the S1 sample. The mortars named S3 and S4 contained more fluid, with the S3 mixture showing the same workability as the reference. These results were attributed to the size of the cuttings—in particular, the shortest fibres showed the highest specific surface which determined an increase in water absorption with consequent reduction in workability. The mixture with perlite (P) showed similar consistency to the control, while the straw/perlite mortars showed intermediate values between bare straw and bare perlite composites. In this respect, the presence of perlite improved the fluidity of the S1 and S2 mortars; the P/S1 and P/S2 flows were, respectively, in the range −30% to −25% with respect to the control. The P/S3 and P/S4 mixtures showed similar consistency to the normalised mortar. It was also observed that the fresh composites showed a sensible decrease in the flow with increase in straw volume due to the increasing water absorption which determined the manufacture of the dry materials, as in the case of the S3B and S3C samples (−60% and −100%, respectively).

### 3.2. Thermal and Acoustic Measurements

**Figure 4:**
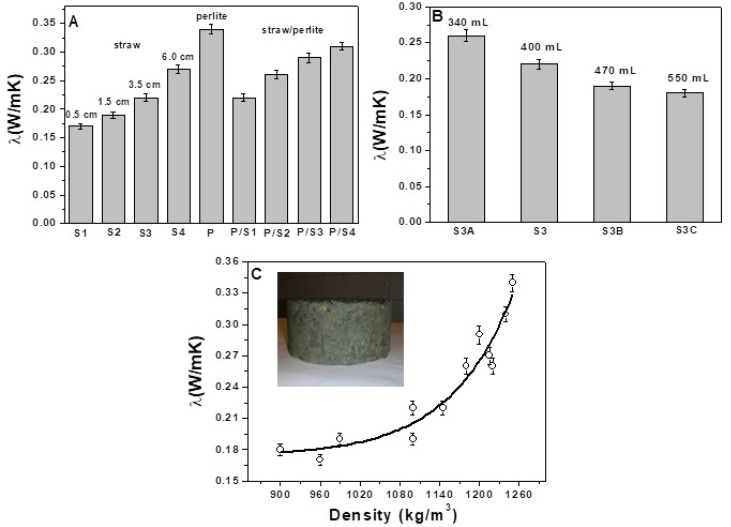
(**A**) Thermal conductivity of the specimens with the same volume of aggregates (400 mL). (**B**) Thermal conductivity of the S3 specimens with different volume of aggregates. (**C**) Exponential increase in the thermal conductivity with density increase. In the inset: image of a sample for thermal detections.

**Figure 5:**
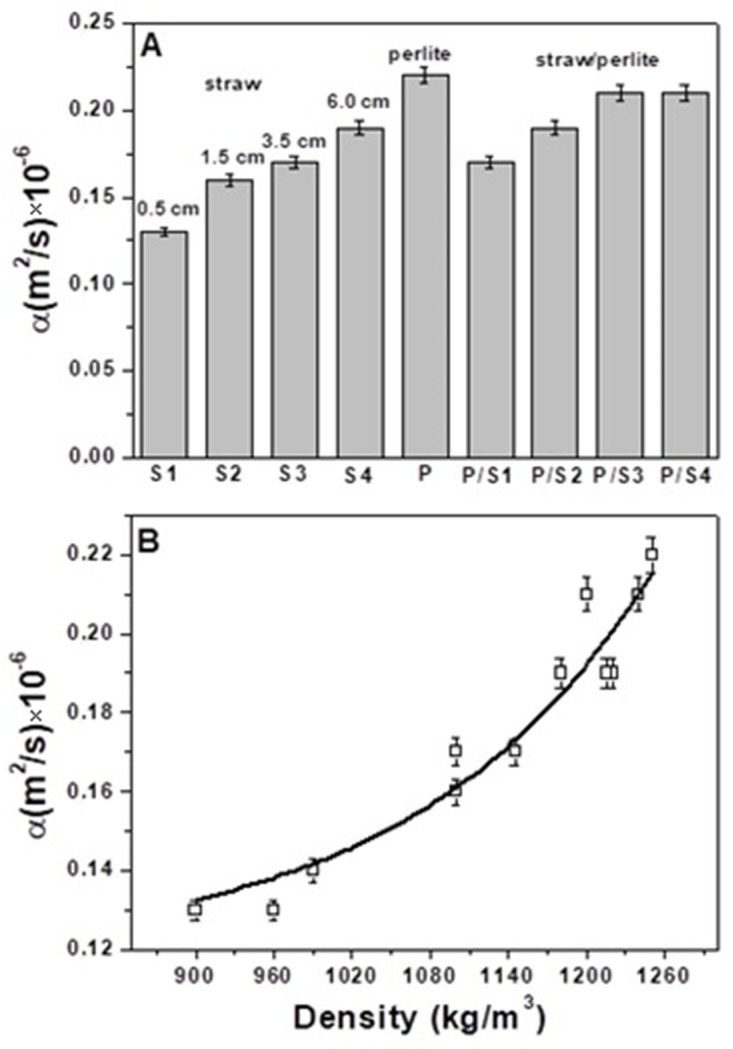
(**A**) Thermal diffusivity of the specimens with the same volume of aggregates (400 mL). (**B**) Exponential increase in the thermal diffusivity with density increase.

The thermal conductivities and diffusivities of the straw mortars were very much lower compared to the sand reference which showed a thermal conductivity in the range of 1.8–2.0 W/mK and a thermal diffusivity in the range of 1.2–1.4 × 10^−6^ m^2^/s. Specifically, the thermal conductivity decrease (%) for the entire set of straw based composites (S1, S2, S3, S4, S3A, S3B and S3C) was in the range of 86–91%, while the thermal diffusivity decrease (%) was in the range of 85–89% (Figure 4 and Figure 5).

**Figure 6:**
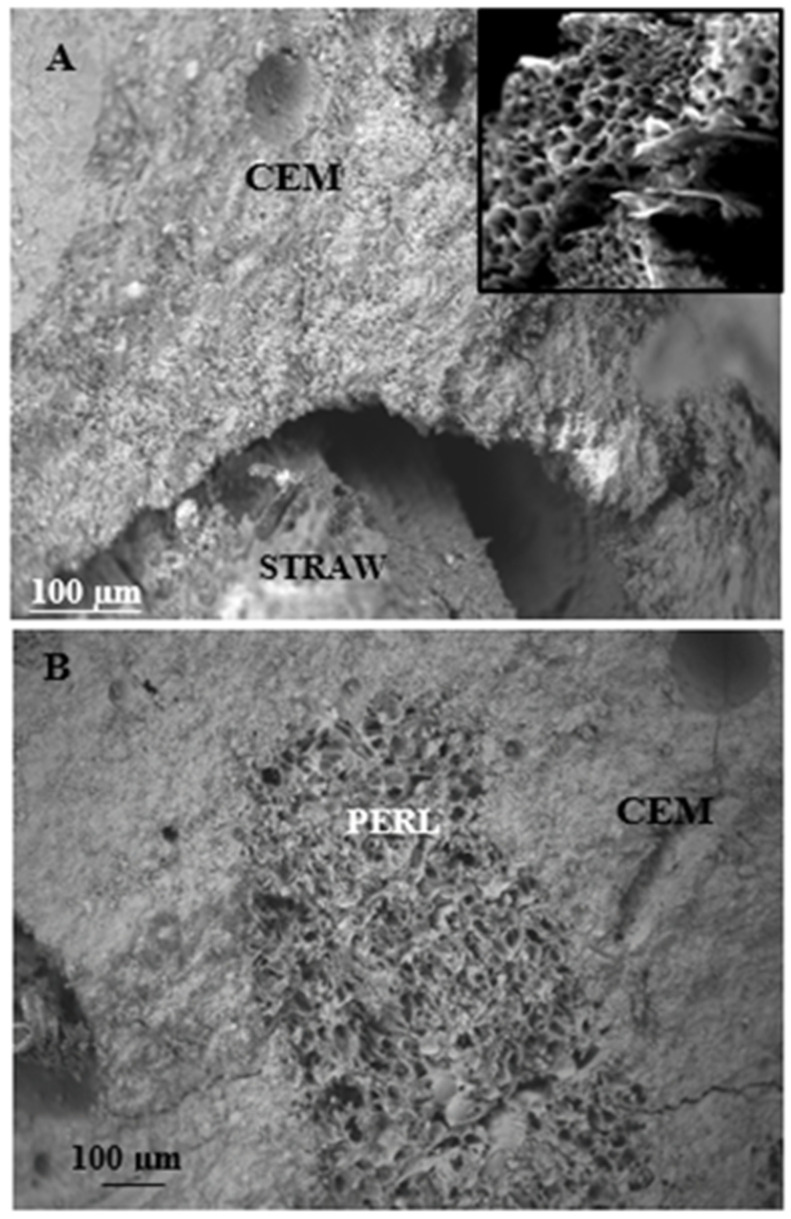
(**A**) SEM image of the cement/straw interface, in the inset: porous structure of the straw. (**B**) SEM image of the cement/perlite interface.

The reduction in thermal conductivity and diffusivity of the straw-containing mortars can be attributed to the hollow lumen structure of the organic aggregate, as observed in the analysis of the cross-section of the straw and can be ascribed to the action of the aggregate in modifying the structure of the mortars (Figure 6A) [29,30,31]. From the SEM detections it can be seen that poor adhesion of the organic straw fibres to the inorganic cement matrix was responsible for the formation of voids at the organic/inorganic interface. For all these reasons, a reduction in the specific mass (in the range of 39–52%) and an increase in the porosity (in the range of 45–54%) of the composites was observed (Table 2) [41].

The generation of voids in the cement matrix contributes to limiting heat transport with increase in thermal insulation [29,30,31]. The composite S1, with the lowest size of fibres, showed the highest thermal insulation (λ = 0.17 W/mK, α = 0.13 × 10^−6^ m^2^/s) at the same volume of aggregate in the mixture, which tended to decrease with increase in straw length (Figure 4A and Figure 5A). This result can be explained by the highest specific surface of this type of fibre which is responsible of the generation of the highest percentage of voids at the interface, together with the increased number of voids attributed to the porous structure of the bare aggregate. In fact, the S1 specimen showed the highest porosity (48%) and the lowest density (960 Kg/m^3^) among the straw composites (S2, S3 and S4, Table 2). This demonstrates the effect of different length fibres on the conglomerates.

The results show that an increase in straw content decreased the thermal conductivity and diffusivity of the composites (Figure 4 and Figure 5). In fact, the S3C sample, with the highest dosage and volume of fibres (550 cm^3^), was characterised by the lowest density (900 kg/m^3^), the highest porosity (48%) and the lowest thermal conductivity (0.18 W/mK) and diffusivity (α = 0.13 × 10^−6^ m^2^/s), with respect to S3B, S3 and S3A composites which were characterised by an increase in the specific mass (990 kg/m^3^, 1145 kg/m^3^, 1220 kg/m^3^, respectively), decrease in porosity (46%, 44%, 40%, respectively) and increase in thermal conductivity (0.19 W/mK, 0.22 W/mK and 0.26 W/mK, respectively) and diffusivity (0.14 × 10^−6^ m^2^/s, 0.17 × 10^−6^ m^2^/s and 0.19 × 10^−6^ m^2^/s, respectively). This result could be due to the increasingly lower encapsulation of the fibres in the cement matrix which increased the voids at the organic/inorganic interface. From the rheological tests, it was also observed that a sensible decrease in the flow of the fresh conglomerates with rise in the straw volume occurred which caused the production of increasingly dry specimens with consequent increase in the porosity of the hardened artifacts. The sample with bare perlite (P) showed the highest value of density (1250 Kg/m^3^) and the lowest porosity (37%) among all the other lightweight samples; the thermal conductivity was in the range of 0.34 W/mK, accordingly, this specimen resulted in the lowest thermal insulating properties. The perlite thermal conductivity was ~50% higher than the value obtained with the S1 sample with a density increase and porosity decrease in the range of ~23%. Figure 6B demonstrates these results; good adhesion of the perlite to the cement paste can be observed due to the beads roughness and the similar chemical compounds in both the mixture components (silicates and aluminates) [70]. Thus, the porosity of this type of mortar was exclusively associated with the closed porosity of the perlite (Figure 2B) and not the presence of empty spaces at the ligand/aggregate interface, as in the case of the straw-based samples. The straw/perlite samples (P/S1, P/S2, P/S3 and P/S4) showed intermediate values of thermal conductivity and diffusivity as a result of the intermediate values of density and porosity. An exponential increase in thermal conductivity and diffusivity was observed with increase in conglomerate density (Figure 4C and Figure 5B).

**Figure 7:**
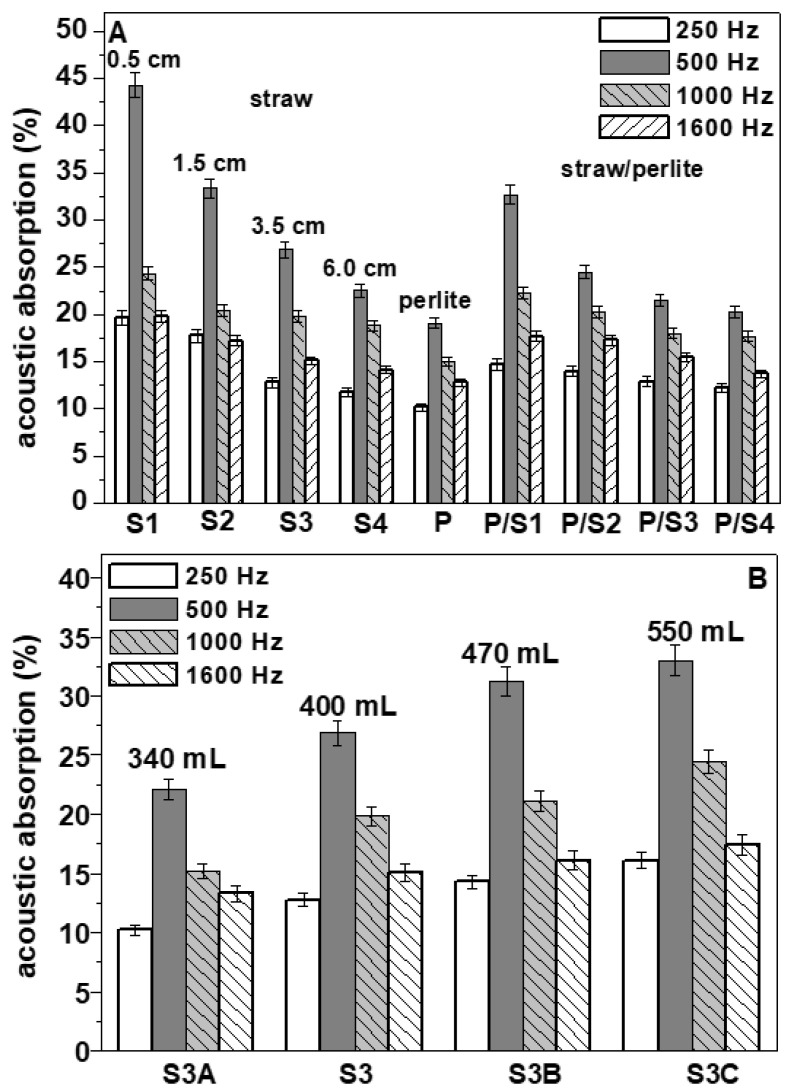
(**A**) Acoustic absorption of the specimens with the same volume of aggregates (400 mL). (**B**) Acoustic absorption of the S3 specimens with different volume of aggregates.

To evaluate the acoustic characteristics of the cellulose-cement composites, the normal incident absorption coefficient (α) was determined. When a sound wave strikes a material, a portion of the sound energy is reflected while a portion is absorbed. This coefficient is the ratio of the absorbed energy to the total incident energy and is determined by the Kundt impedance tube [78]. It is calculated as:

α = 1 − ρ(1)

where ρ is the reflection coefficient of the acoustic energy, expressed as the ratio between the reflected and the incident energy. Figure 7 shows the acoustic absorption data carried-out at 250, 500, 1.000 and 1.600 Hz. Specifically, the percentage increase for the entire set of straw-based composites (S1, S2, S3, S4, S3A, S3B and S3C) was in the range of 10–54% at 250 Hz, 77–89% at 500 Hz, 27–54% at 1000 Hz and 54–70% at 1600 Hz with respect to the control which showed the following results: 9% at 250 Hz, 5% at 500 Hz, 11% al 1000 Hz and 6% at 1600 Hz. As previously reported, this result can be ascribed to the intrinsic porosity of the natural aggregate (inset Figure 6A) and to the action of straw in modifying the structure of the mortars by creating pores in the cement matrix (Figure 6A) with consequent reduction in the specific mass [29,30,31]. Accordingly, in these specimens, straw-induced formation of voids occurred where acoustic energy was likely to be attenuated, in particular, at 500 Hz where, after resonance phenomena, the closed cavities might play a major role [34,62,79,80]. The S1 sample, with the lowest size of fibres, at the same volume of aggregate in the mixture, showed the highest acoustic absorption at all the frequencies, with specific reference to the 500–1.000 Hz range. This value decreased with increase in straw length (S2, S3 and S4 specimens) because of the decrease in composite porosity (Figure 7A and Table 2).

The increase in the straw content and volume determined an increase in acoustic absorption (Figure 7B). The S3C sample, with the highest dosage and volume of fibres, showed at 500 and 1000 Hz values in the range of 33% and 24%, respectively, while the S3B (31% and 21%, respectively), the S3 (27% and 20%, respectively) and the S3A (22% and 15%, respectively) were characterised by increasingly lower values of α at lower straw content. This result can be explained by the highest porosity of S3C with respect to S3B, S3 and S3A composites which were characterised by a decrease in porosity with increase in the straw volume in the matrix. The sample with bare perlite (P) showed the lowest acoustic absorption because of the highest value of density (1250 Kg/m^3^) and the lowest porosity (37%) among all the other lightweight samples. The straw/perlite samples (P/S1, P/S2, P/S3 and P/S4) showed intermediate values of α as a result of the intermediate values of density and porosity.

### 3.3. Mechanical Tests

**Figure 8:**
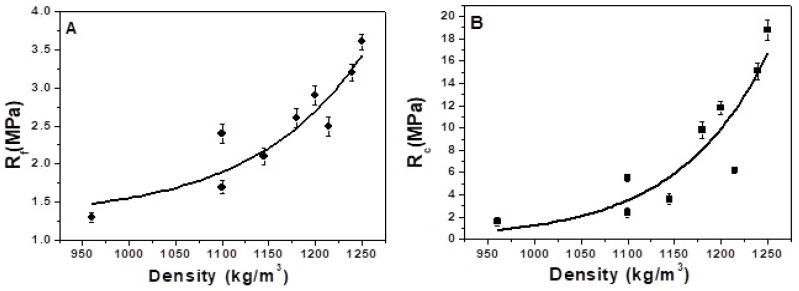
(**A**) Flexural and (**B**) compressive strengths of the S, P and S/P mortars with the same volume of aggregate (400 mL) as a function of the composite density (28 days ageing).

Flexural and compressive strengths (at 28, 60, and 90-days ageing) of the samples are shown in Table 3 and Figure 8A,B. A general increase between 24 and 60 days and a final stabilisation between 60 and 90 days was observed. Moreover, Figure 8A,B show an exponential increase in mechanical resistance with the specific mass of the conglomerates.

From a general overview of data referring to straw-based composites (S1, S2, S3, S4, S3A, S3B and S3C), a sensible decrease in the mechanical performances with respect to the sand reference was observed which showed flexural and compressive resistances at 28-days ageing in the range of 8.5–9.0 MPa and 48–50 MPa, respectively.

The conglomerates with bare straw aggregates showed flexural resistances in the range of 1.3–2.5 MPa and compressive resistances in the range of 1.6–6.2 MPa. The decrease in mechanical strength can be attributed to the already mentioned low density of the straw fibres compared to the cement paste and to lack of adhesion of the organic aggregate to the cement paste [22,30,31]. Straw particles showed lower stiffness than the surrounding cement paste; accordingly, under loading, cracks initiated around the straw accelerated the failure in the matrix. The increase in porosity associated with the voids at the fibre–matrix interfaces further affected the lowering of mechanical performances.

**Figure 9:**
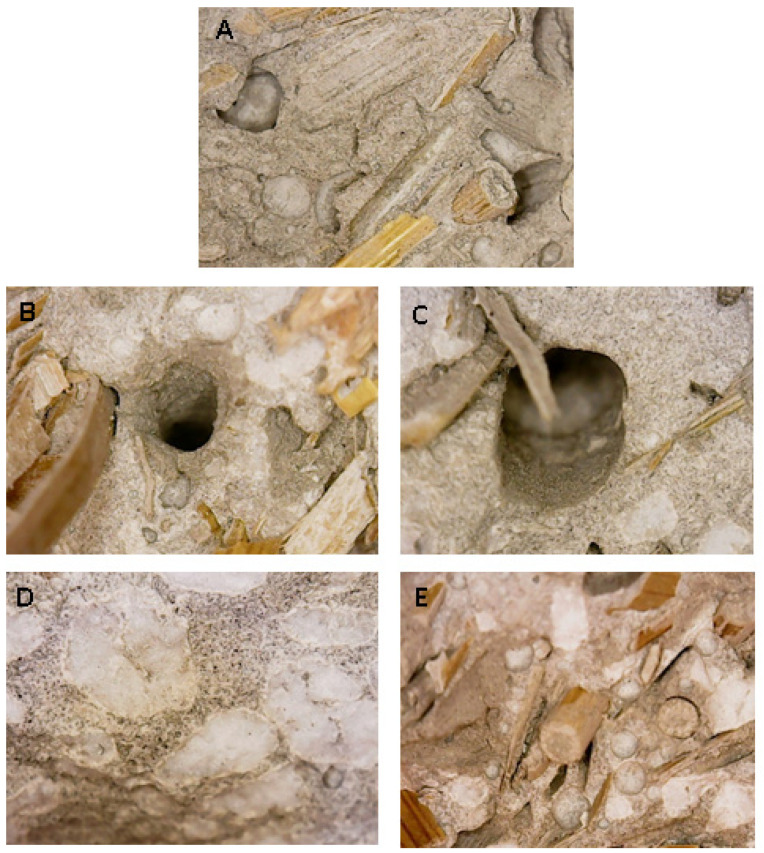
Failure plane of the (**A**) S1 sample, (**B**,**C**) holes caused by the pull-out of the straw, sections of the (**D**) P sample and of the (**E**) P/S1 sample.

Figure 9A shows that these types of composites presented a good aggregate distribution and straw–matrix compatibility with the cement paste around and inside the fibres [21].

Moreover, it was observed that an increase in the resistances, with increase in straw length at the same volume of aggregate in the mixture, was also associated with increasing density and decreasing porosity of the conglomerates. The S1, S2, S3 and S4 specimens showed flexural strengths corresponding to 1.3 MPa, 1.7 MPa, 2.1 MPa and 2.5 MPa, respectively, while they showed compressive strengths corresponding to 1.6 MPa, 2.4 MPa, 3.6 MPa and 6.2 MPa, respectively.

**Figure 10:**
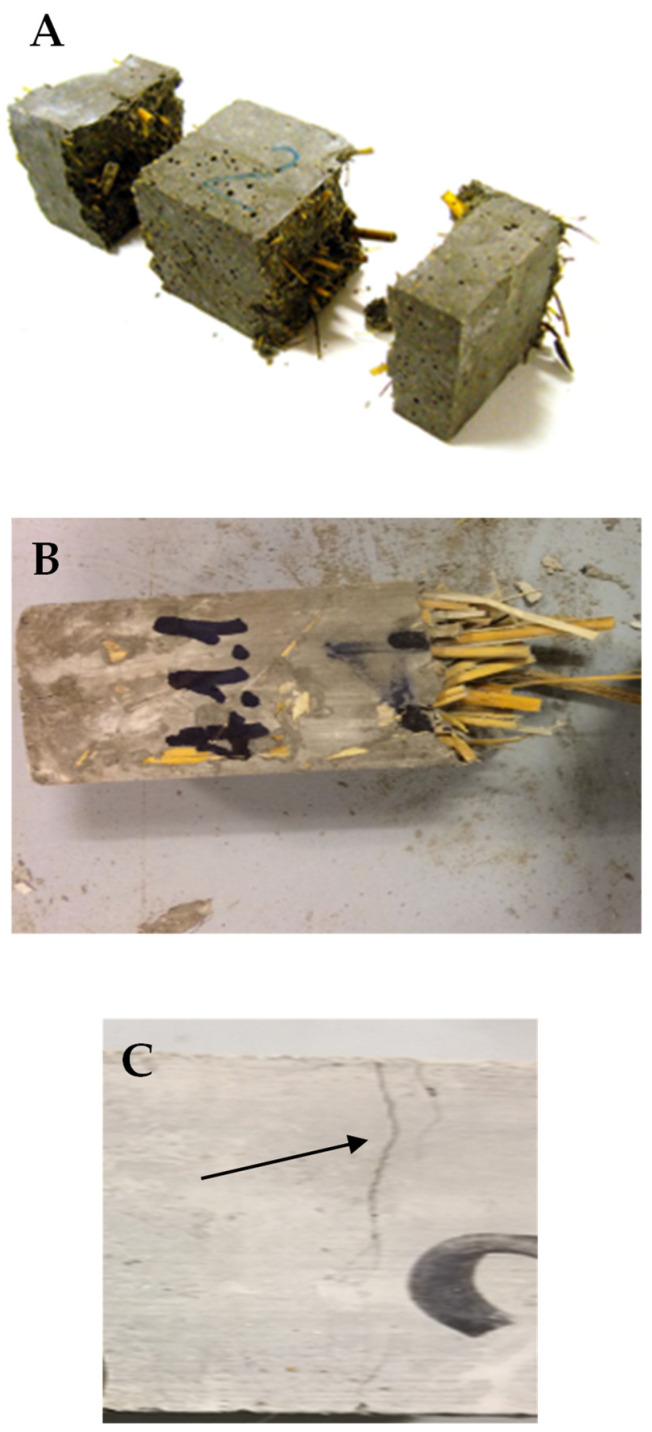
Fibres horizontal arrangement in (**A**) P/S3 and (**B**) S4 specimens. (**C**) Discrete cracks after rupture in the straw specimens (evidenced by the arrow), with the two parts of the sample still connected by the organic aggregate.

The increase in straw content/volume in the composites decreased the flexural and compressive strengths, as shown in Table 3. Flexural strength at 28 days decreased from 2.5 MPa to 1.7 MPa, while compressive strength decreased from 6.2 MPa to 2.4 MPa for composites containing from 340 mL to 550 mL straw volume, respectively. This result was associated with decrease in encapsulation of the fibres in the cement matrix at increasing straw volume which increased the porosity of the samples. During the flexural load application, the interference of the nearby fibres induced a loss of the straw/matrix bonding. Accordingly, the fibres were pulled out from the matrix and considerable energy was lost from the system in the form of frictional energy [22,23,66]. After the breakage, a separation of the two parts of the samples was not observed, with the two semi-prisms of the samples still connected by the fibres. This effect was associated with straw tensile strength [30,81] and was affected by the density decrease and by the structure of the fibres which showed a horizontal arrangement during the mortar preparation [30] that was confirmed after final separation of the two semi-prisms. In this respect, it was observed that the fibres were encapsulated in one of the two parts while pulling out from the other. Figure 9B,C show the holes derived from the pull-out of the cellulose fibres from the matrix, while Figure 10A,B show the horizontal arrangement of the aggregate after rupture [30]. Figure 10C shows that a real collapse of the specimen was not observed after breakage but only cracks ascribed to the plastic behaviour, mostly detected at high straw content.

The sample with bare perlite (P) showed the highest mechanical resistances (R_f_ = 3.5 MPa and R_c_ = 18.8 MPa) due to the higher stiffness of the silica aggregate with respect to straw and to the good adhesion to the ligand paste at the interface which increased the density of the mortar. Figure 9D shows an image of the surface of this composite after rupture and its good particle distribution. The straw/perlite samples (P/S1, P/S2, P/S3 and P/S4) showed intermediate values as a result of the intermediate values of density and porosity. Figure 9E shows that these composites, with a mixture of aggregates, also presented a good particle/fibre distribution and straw–matrix compatibility with the cement paste around and inside the fibres.

From a general point of view, the P/S3 and P/S4 samples can be considered interesting composites with good workability (similar to the normalised mortar), low thermal conductivities (~0.30 W/mK) and good mechanical properties (R_f_ = 2.9 MPa and R_c_ = 11.8 MPa, in the case of the P/S3 specimen, R_f_ = 3.2 MPa and R_c_ = 15.1 MPa, in the case of the P/S4 specimen).

**Figure 11:**
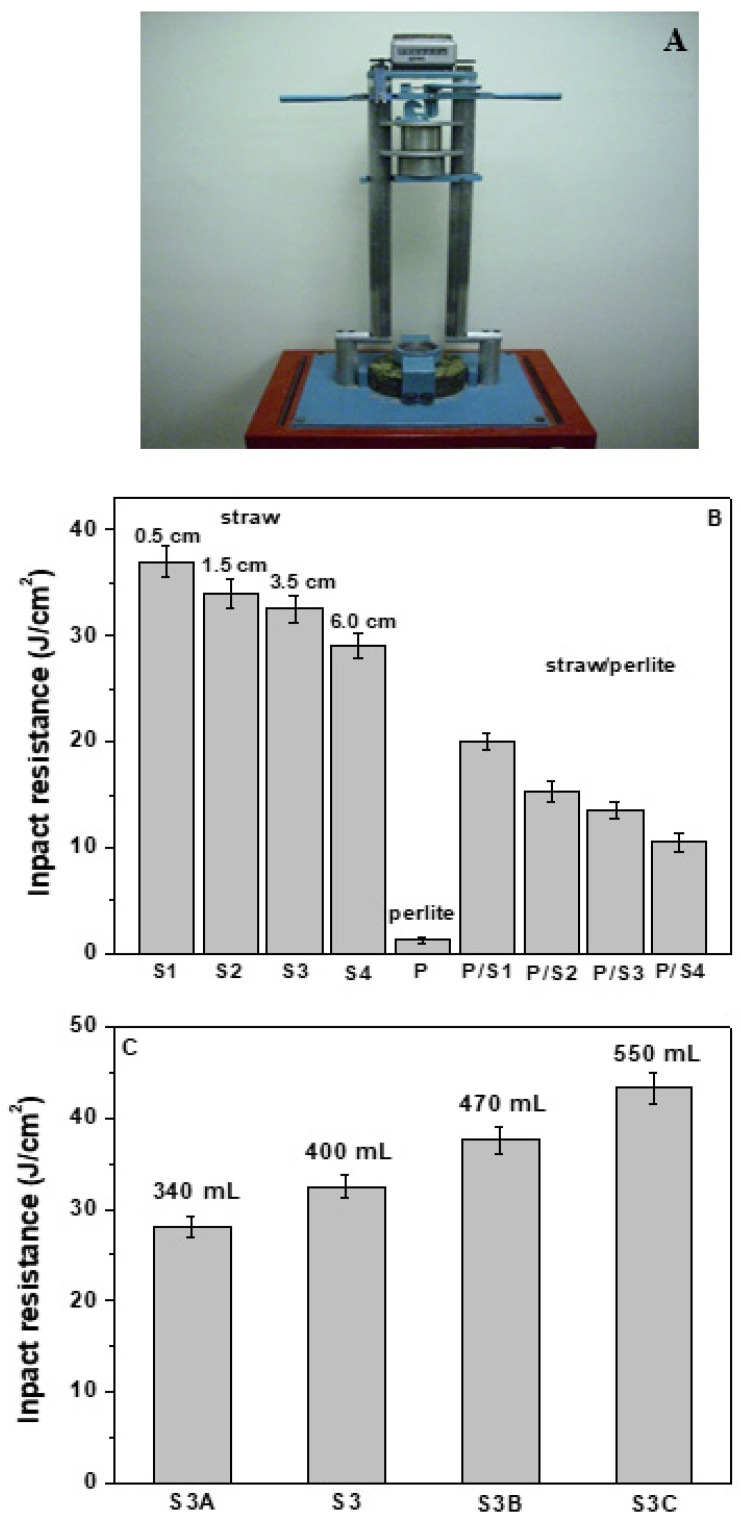
(**A**) Impact resistance apparatus. (**B**) Impact resistance of the specimens with the same volume of aggregates (400 mL). (**C**) Impact resistance of the S3 specimens with different volume of aggregates.

**Figure 12:**
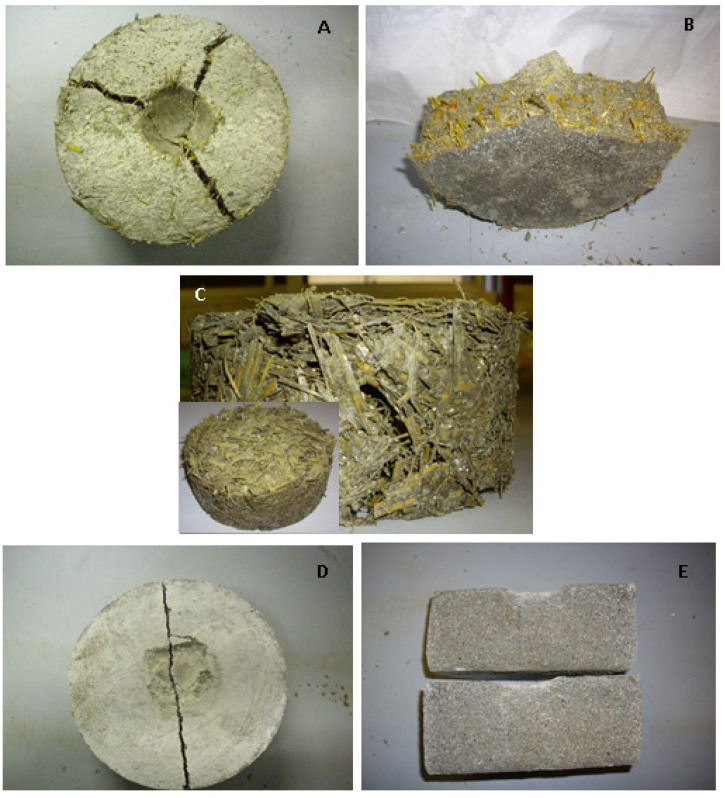
Samples after the impact test. (**A**,**B**) S3 sample, (**C**) S3C sample, (**D**,**E**) perlite sample.

The impact compression tests, obtained with the experimental apparatus of Figure 11A, showed that the straw samples (S1, S2, S3, S4, S3A, S3B and S3C) were characterised by high energy absorption capacity, with specific reference to the S1 (Figure 11B) and S3C (Figure 11C) samples. The toughness of these composites was improved as fibre length decreased and as fibre volume increased (increasingly low specific mass) and was characterised by a deep groove before complete failure (Figure 12A) [82,83]. The horizontal arrangement after pull-out was confirmed, as shown in Figure 12B relative to the S3 sample [30].

As observed in the flexural strength tests, a separation of the parts of the sample was not observed because the parts were still connected by the fibres (Figure 12A,B). The high straw tensile strength affected the formation of cracks instead of an evident breakage [81] and this effect was observed particularly in samples with high straw dosage, as in the case of the S3C specimen (Figure 12C). 

The perlite sample (P) was fragile and breakage occurred after a few blows due to the presence of the brittle aggregate (Figure 12D,E), while the samples with 50% of straw and 50% of perlite represented a compromise between energy absorption capacity attributed to the natural fibres and mechanical resistance attributed to the inorganic aggregate (Figure 11B).

### 3.4. Stability of the Composites

From visual inspections and optical microscopical observations, the straw surface structure, evidenced in Figure 9 and Figure 10B, was similar to the pristine (inset Figure 1A) and also retained its original color, both indications of negligible degradation occurring after chemical interaction between the fibres and the ligand paste [30]. Specifically, Figure 9E is an optical microscope image of the P/S1 sample after more than one year from the breakage and after curing at room temperature and at 75–80% relative humidity.

**Figure 13:**
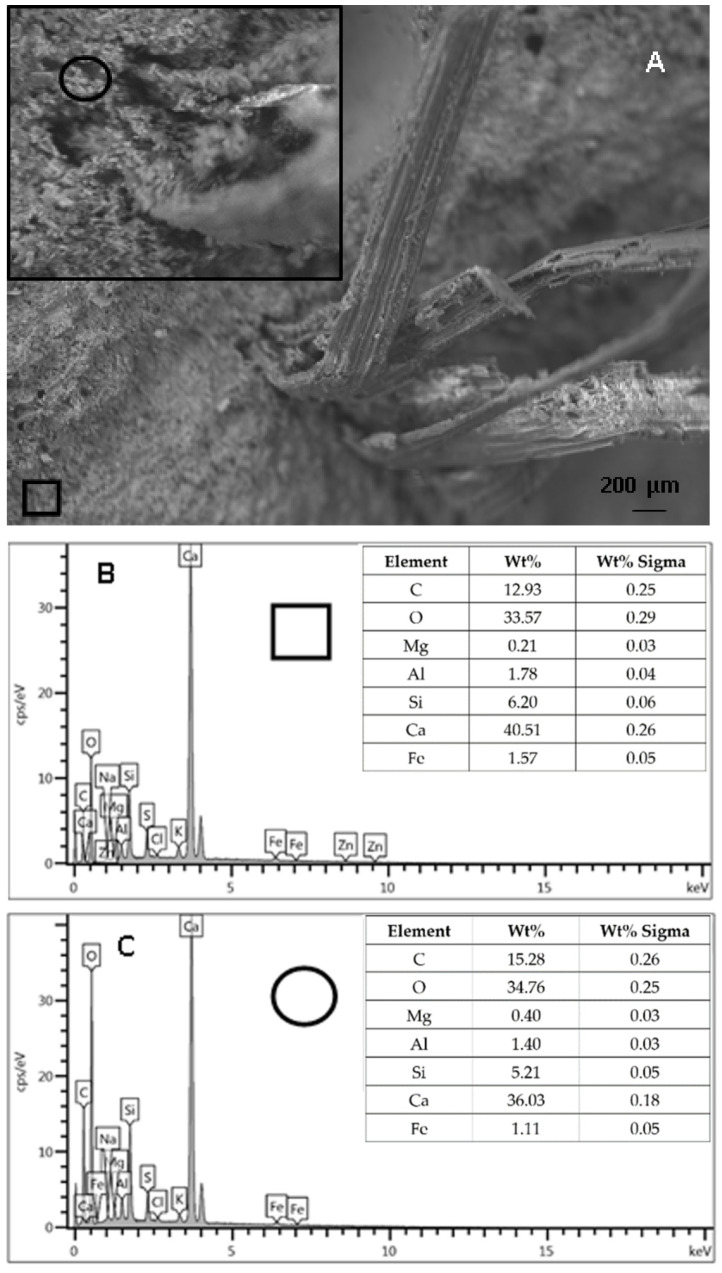
(**A**) SEM image of the cement/straw system with square spot showing the cement paste far from straw, in the inset: magnification of the cement paste close to straw and represented by circular spot. (**B**) EDX spectrum relative to the square spot (far from straw). (**C**) EDX spectrum relative to the circular spot (close to straw).

It has been reported that the degradation of vegetable fibres is associated with the presence of calcium hydroxide in the matrix at the basic pH of the cement paste [42,45,46] due to the dissolution of water-soluble plant compounds. Accordingly, the concentration of soluble calcium compounds was much higher than the concentration of silicon compounds in the areas of the cement paste close to the straw fibres. As a result, drawbacks, such as shift of the setting time, delay in mechanical strength development, and stiffness increase were evidenced [30]. Figure 13B,C show EDX analyses of the cement paste close to the straw fibres and far from the fibres (Figure 13A) in order to have a chemical characterisation of two different zones, specifically the Ca/Si ratio [84,85]. The sample was withdrawn in a straw-based specimen cured for more than one year in a humid environment (75–80%). In the present case, the chemical compositions were similar to the Ca/Si ratios, which is a further indication of negligible degradation of these composites together with the optical observations and the stable values of mechanical resistance in the range of 28–90 days. The reported results can be considered preliminary investigations on the features of the composites during time and not durability studies, but they can be very useful for the preparation of sustainable indoor cement artifacts based on cellulose fibres and perlite.

## 4. Conclusions

Eco-sustainable cement conglomerates containing untreated wheat straw and perlite beads as aggregates were prepared and characterised by rheological, thermal, acoustic, mechanical and microstructural measurements. A complete replacement of the conventional sand aggregate was carried out with straw cuttings of different length and dosage and with perlite beads. Composites with bare straw (S), perlite (P), and with a mixture of organic and inorganic aggregates (P/S), were characterised and compared with the properties of normalised sand mortar.

From the rheological characterisation, the fibres with the lowest length showed the highest specific surface which influenced water absorption with consequent reduction in workability (e.g., S1 and S2 samples, extremely dry). With an increase in straw length an increase in fluidity (S3 and S4) was observed because of the lower water absorption with respect to the former samples (i.e., plastic behaviour). Fresh composites also showed a sensible decrease in flow with increase in straw volume, with S3C extremely dry. The mixture with perlite (P) showed similar consistency to the control, while the straw/perlite mortars showed intermediate values between bare straw and bare perlite composites. All these samples had good workability useful for plastic castings. Specifically, the conglomerate with bare perlite and the P/S3 and P/S4 mixtures showed similar consistency to the control.

The thermal insulation of the straw mortars was extremely high compared to the sand reference. Specifically, the thermal conductivity quenching for the entire set of the straw-based composites was in the range of 86–91%. It was ascribed to the hollow lumen structure of the organic aggregate and to the poor adhesion of the straw fibres to the cement matrix which determined a reduction in the specific mass (39–52%) and an increase in porosity (45–54%) with respect to the reference. The results showed an enhancement of the thermal insulation with decrease in straw length and with increase in straw volume due to the increase in porosity of the composites. Lower thermal insulation was obtained with the mixtures of aggregates (P/S) because of the reduction in porosity associated with the presence of perlite which showed good adhesion to the cement paste.

The acoustic absorption of the straw mortars was extremely high compared to the sand reference, especially in the 500–1000 Hz range. These results were ascribed to the high porosity of these composites and showed an enhancement with decrease in straw length and with increase in straw volume.

A sensible decrease in mechanical performance with respect to the sand reference was obtained and the values increased with the length of the straw and decreased with the straw dosage. The addition of expanded perlite to the mixture allowed mortars to be obtained with an improvement in mechanical strength and negligible modification to thermal properties. Straw mortars showed discrete cracks after failure without separation of the two parts of the specimens due to the aggregate tensile strength.

The impact compression tests showed that the straw samples were characterised by high energy absorption capacity, with specific reference to the S1 and S3C samples. These parameters were improved as fibre length decreased and as fibre volume increased (increasingly low specific mass) and were characterised by a deep groove before complete failure.

Microscopical observations, after more than one year curing in 75–80% humidity, revealed negligible degradation of these composites, while mechanical tests showed stable values in the range of 28–90 days.

Based on the physical and mechanical results, non-structural indoor applications (e.g., panels, plasters) may be considered for these lightweight composites, with specific reference to conglomerates made of straw and perlite which can be considered a good compromise between thermo-acoustic and mechanical properties. It is important to underline the environmental advantages related to the recycling of agricultural waste adopting a safe and environmentally friendly process with respect to the circular economy.

## Figures and Tables

**Table 1:** Mortars composition.

Sample	Type	Cement (g)	Water (cm^3^)	Perlite Weight (g)	Perlite Volume (cm^3^)	Straw Volume (cm^3^)	Straw Weight (g)
S1	straw 0.5 ± 0.3 cm	450	225	0	0	400	55
S2	straw 1.5 ± 0.3 cm	450	225	0	0	400	44
S3	straw 3.5 ± 0.3 cm	450	225	0	0	400	34
S3A	straw 3.5 ± 0.3 cm	450	225	0	0	340	29
S3B	straw 3.5 ± 0.3 cm	450	225	0	0	470	40
S3C	straw 3.5 ± 0.3 cm	450	225	0	0	550	47
S4	straw 6.0 ± 0.4 cm	450	225	0	0	400	25
P	perlite 3–4 cm	450	225	42	400	0	0
P/S1	perlite 3–4 cm/straw 0.5 cm	450	225	21	200	200	27.5
P/S2	perlite 3–4 cm/straw 1.5 cm	450	225	21	200	200	22
P/S3	perlite 3–4 cm/straw 3.5 cm	450	225	21	200	200	17
P/S4	perlite 3–4 cm/straw 6.0 cm	450	225	21	200	200	12.5

**Table 2:** Density and porosity of the mortars.

Sample	Density (kg/m^3^)	Porosity (%)
S1	960	48
S2	1100	46
S3	1145	44
S3A	1220	40
S3B	990	46
S3C	900	48
S4	1215	41
P	1250	37
P/S1	1100	44
P/S2	1180	42
P/S3	1200	40
P/S4	1240	40

**Table 3:** Flexural and compressive strengths of the S, P and S/P mortars at 28, 60 and 90-days curing.

Sample	Density (kg/m^3^)	R_f_ (MPa) 28 Days	R_f_ (MPa) 60 Days	R_f_ (MPa) 90 Days	R_C_ (MPa) 28 Days	R_c_ (MPa) 60 Days	R_c_ (MPa) 90 Days
S1	960	1.3	1.7	1.6	1.6	2.0	1.9
S2	1100	1.7	2.0	2.1	2.4	2.7	2.8
S3	1145	2.1	2.2	2.3	3.6	3.7	3.5
S3A	1220	2.5	2.6	2.4	4.1	4.3	4.3
S3B	990	1.9	2.0	2.3	3.1	3.4	3.3
S3C	900	1.7	2.0	2.0	2.4	2.5	2.7
S4	1215	2.5	2.8	2.6	6.2	6.2	6.4
P	1250	3.5	4.3	4.5	18.8	19.3	19.7
P/S1	1100	2.4	2.7	2.6	5.5	5.6	5.5
P/S2	1180	2.6	2.9	3.0	9.8	10.1	10.4
P/S3	1200	2.9	3.3	3.2	11.8	11.8	11.9
P/S4	1240	3.2	3.6	3.8	15.1	15.3	15.2

## Data Availability

Data sharing not applicable.
